# Recent Advances in Lipid-Based Nanosystems for Gemcitabine and Gemcitabine–Combination Therapy

**DOI:** 10.3390/nano11030597

**Published:** 2021-02-27

**Authors:** Saffiya Habib, Moganavelli Singh

**Affiliations:** Nano-Gene and Drug Delivery Group, Discipline of Biochemistry, School of Life Sciences, University of KwaZulu-Natal, Private Bag X54001, Durban 4000, South Africa; saffiyahabib@gmail.com

**Keywords:** lipids, nanosystems, gemcitabine, drug delivery, cancer

## Abstract

The anti-metabolite drug gemcitabine is widely used for the treatment of a variety of cancers. At present, gemcitabine is administered as a hydrochloride salt that is delivered by slow intravenous injection in cycles of three or four weeks. Although regarded as a ‘front-line’ chemotherapeutic agent, its efficacy is hampered by poor target cell specificity, sub-optimal cellular uptake, rapid clearance from circulation, the development of chemoresistance, and undesirable side-effects. The use of organic, inorganic, and metal-based nanoparticles as delivery agents presents an opportunity to overcome these limitations and safely harness optimal drug efficacy and enhance their therapeutic indices. Among the many and varied nano delivery agents explored, the greatest body of knowledge has been generated in the field of lipid-mediated delivery. We review here the liposomes, niosomes, solid lipid nanoparticles, nanostructured lipid carriers, exosomes, lipid-polymer hybrids, and other novel lipid-based agents that have been developed within the past six years for the delivery of gemcitabine and its co-drugs.

## 1. Introduction

Gemcitabine (2′,2′-difluoro-2′-deoxycytidine; dFdC) is a deoxycytidine analogue marketed as the hydrochloride salt, Gemzar^®^ [1]. Originally investigated as an anti-viral agent, gemcitabine was later developed for the treatment of cancer [2]. Gemzar^®^, in combination with another chemotherapeutic agent, cisplatin, was approved by the Food and Drug Administration in 1996 for the treatment of inoperable stage III or IV non-small cell lung cancer [3]. It has since been applied to the treatment of a wide range of solid tumors, usually in combination with other drugs [3,4,5,6].

Currently, gemcitabine is introduced intravenously in three or four-week cycles [7]. Cells internalize gemcitabine via plasma membrane-bound human nucleoside transporter proteins [8]. Within the cell, the dFdC prodrug is metabolized to the mono- (dFdCMP), di- (dFdCDP), and tri-phosphate (dFdCTP) forms [9,10,11]. The incorporation of dFdCTP into DNA inhibits replication by terminating DNA chain elongation. While this is the major mode of action of the drug [12], gemcitabine also acts by inhibiting the activity of enzymes implicated in the metabolism of deoxynucleotides [13,14], and by inducing apoptosis via caspase signaling [15,16].

Although gemcitabine is considered a first-line chemotherapy drug, it is by no means perfect. Gemcitabine treatments are plagued by issues such as low drug sensitivity and unpleasant side effects [17]. Furthermore, the drug is rapidly deaminated in the blood to inactive 2′, 2′-deoxyfluorouridine and excreted in the urine. An additional concern is the development of chemoresistance with the loss of transporter proteins and kinases required for phosphorylation in some cancers [18]. Evidently, the full anticancer potential of the drug can only be profitably and safely harnessed with much improvement. Here, two strategies have emerged. One is to chemically modify the drug itself [1]. The other is to develop an appropriate drug delivery platform [19].

**Figure 1:**
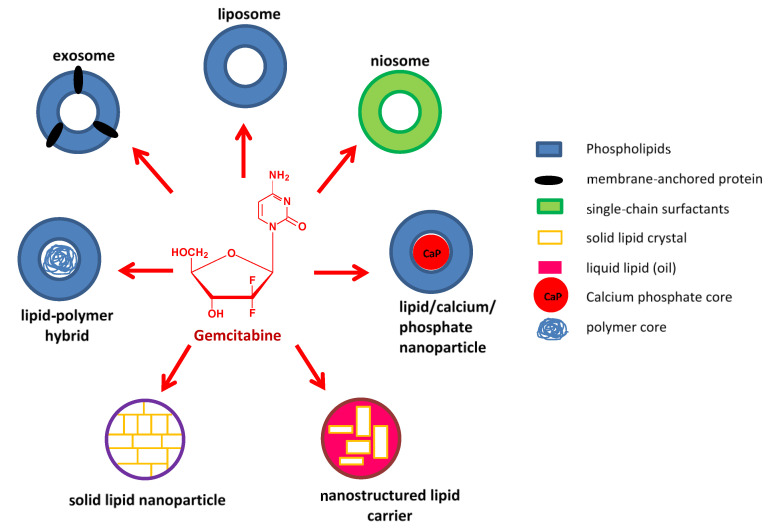
Illustration of some lipid-based nanocarriers being used in gemcitabine delivery.

In this regard, the idea of associating gemcitabine with nanoparticles is gaining impetus. Nanocarriers possess unique physicochemical and biological properties imbuing them with multifunctional abilities than can allow for the simultaneous delivery of multiple drugs with improved retention, controlled release, and effective delivery of payloads specifically to target cells; thereby reducing the overall dose and minimizing side effects [20,21]. Metallic nanoparticles have become increasingly popular due to their inherent optical features and relative non-toxicity [22,23,24], but they are still to be fully explored. Lipid-based nanostructures represent the earliest, most widely studied, and continually advancing nano delivery agents; and have been extensively investigated for the delivery of gemcitabine and its co-drugs. The current review has attempted to feature advances in gemcitabine- and gemcitabine-combination lipid-based nanosystems that have come to the fore between 2015 and 2021. Figure 1 illustrates some of the lipid-based carriers that will be discussed.

## 2. Liposomes

**Figure 2:**
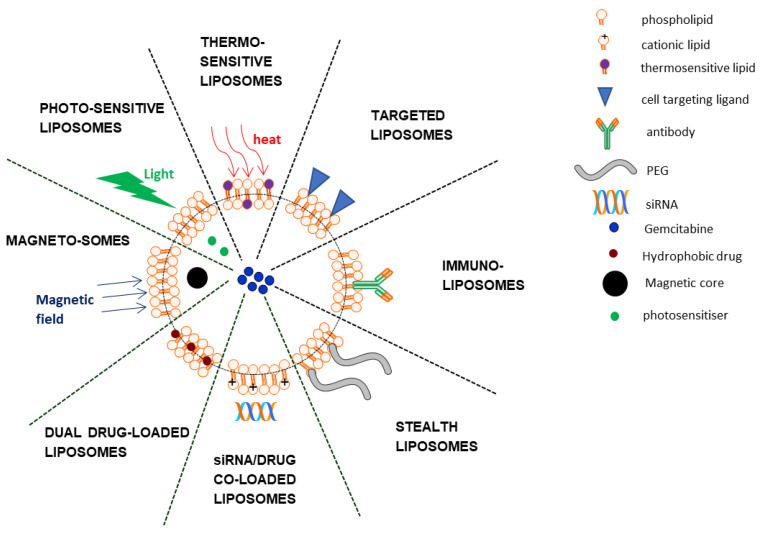
Schematic representation of some liposomal gemcitabine delivery systems.

Arguably the most famous lipid-based nanostructures are the liposomes. These spherical lipid vesicles are comprised of a phospholipid bilayer surrounding an aqueous core within which various substances may be contained. It is this property that is exploited for the loading of drugs such as gemcitabine. Moreover, liposomes are biocompatible, have slow-release profiles, and can be chemically modified to extend circulation time and/or target cancer cells, making them suitable nanocarriers [25]. Not surprisingly, liposomes comprise the largest nanoplatform for the delivery of gemcitabine. Figure 2 provides a summary of liposomal gemcitabine systems discussed in this review.

Tamam and co-workers [26] reported a combination of loading methods to yield an unprecedented high drug loading capacity of gemcitabine into cholesterol-based liposomes. Liposomal gemcitabine demonstrated better stability, sustained drug release, enhanced cellular uptake, and cancer cell death when compared with the free drug. Liposomal gemcitabine has also shown extended plasma time and lower clearance. As in these studies, several groups [27,28,29,30,31,32,33,34] have modified gemcitabine liposomes with the steric stabilizing agent poly(ethylene) glycol (PEG) to inhibit adverse interactions with serum and mask the liposome from immune recognition. 

However, stealth modification is not without its drawbacks. The PEG-shield is known to inhibit cellular uptake, endosomal escape of liposomes, and release of its contents. For this reason, the pH-sensitivity of gemcitabine liposomes is an important feature [35]. Xu and co-workers [36] reported that post-insertion of PEG chains enhanced pH-sensitivity of gemcitabine liposomes as opposed to their pre-inserted counterparts, possibly due to reduced viscosity on the inner liposomal bilayer and increased bilayer fluidity. As an alternative, acid-labile PEG-lipids were introduced to enhance the efficiency of endosomal escape without compromising stealth features [30]. PEG-cleavable pH-sensitive gemcitabine liposomes showed higher accumulation in pancreatic cancer xenografts than liposomes without the cleavable lipid [37]. While most in vivo studies aim for systemic introduction of liposomal gemcitabine, Gandhi and co-workers [38] used a lyophilization technique to prepare a dry, inhalable powdered form of liposomal gemcitabine for the treatment of lung cancer.

Besides stealth modification, the introduction of ligand-targeting motifs on the surface of the gemcitabine-loaded liposome to permit cancer cell recognition and improve uptake is also a common feature. In this regard, the use of antibodies [31,39,40,41], immune adjuvants [42], folic acid [43], hyaluronic acid [44,45], and peptides [46] has been documented. As an example, anionic liposomes encapsulating gemcitabine for breast cancer treatment were modified with the RGD (alanyl glycyl aspartic acid) peptide that binds to the α_V_β_3_ integrin that is overexpressed by these cells. The treatment inhibited tumor growth more effectively than unmodified gemcitabine liposomes and the pure drug, without toxicity in normal cells [47].

The use of physical agents has proven useful to trigger drug release and promote the deposition of gemcitabine liposomes in tumor tissues. Mild hyperthermia heating assists liposomal gemcitabine delivery by increasing vascular permeability in solid tumors and by encouraging the release of the drug [48]. Thermally-active gemcitabine loaded liposomes were evaluated with respect to pancreatic cancer [48,49]. As an example, Kirui and colleagues [50] used gold nanorod mediated mild hyperthermia conditions to drive gemcitabine loaded distearoylphosphocholine liposomes into tumors. The overall enhancement of drug delivery resulted in a reduction in the dose for efficient tumor growth inhibition. Liposomes can also serve as cavitation agents for ultrasound-mediated delivery—thermally-activated liposomes containing gemcitabine reduced tumor viability in murine models with the application of ultrasound [33]. The application of light represents another mechanical stimulus to promote drug delivery through the incorporation of photosensitizers. A water-soluble photosensitizer co-loaded with gemcitabine in pegylated liposomes enabled near-infrared-mediated drug release that was further modulated by dioleoylphosphatidylethanolamine/cholesterol-mediated membrane fluidity of the liposomes [34].

Furthermore, Kim and co-workers introduced a photosensitizer-conjugated lipid into the bilayer of gemcitabine loaded liposomes, which gave encouraging results in a biliary tract cancer model [27]. Drug release from liposomes can also be controlled by applying an alternating magnetic field to introduce magnetic elements. Magnetite nanoparticle cores and gemcitabine were co-encapsulated by a phospholipid bilayer to give magnetoliposomes. The prepared carrier met the physicochemical criteria for systemic delivery and released 70 % of the drug with 5 min exposure to the magnetic field [51]. 

Enhanced anticancer effects were elicited when liposomal gemcitabine was applied in conjunction with “tumor priming” strategies. Such methods entail altering the tumor microenvironment to enhance the activity of therapeutics and overcome the resistance it may pose. Hylander and co-workers [52] administered Apo2L/TRAIL, a recombinant form of TRAIL (tumor necrosis factor-related apoptosis-inducing ligand), to induce apoptosis, reduce solid stress and interstitial fluid pressure, and condition tumors to liposomal gemcitabine in a patient-derived xenograft model.

Synergistic effects have been observed when gemcitabine was co-loaded in liposomes with other drugs such as cisplatin [53], paclitaxel [39,54], docetaxel [55], doxorubicin [40], bevacizumab [41], and clofazimine [56]. As an example, gemcitabine and cisplatin were co-loaded into a liposome that was modified with a synthetic thermo-responsive polymer. These liposomes demonstrated specific hydrophobic interactions with the membranes of pancreatic cancer cells above the temperature transition of the formulations. Moreover, liposomes resulted in a greater than a 10-fold improvement of the IC_50_ of both drugs in a temperature-dependent manner [53]. In an alternative co-delivery strategy, Herceptin was conjugated to gemcitabine loaded thermosensitive immunoliposomes for delivery to breast cancer cells [57]. In another study, gemcitabine and oxaliplatin were separately loaded into magnetoliposomes. In animal models of breast cancer, tumor inhibition was observed only when liposomes were combined for treatment [58]. Liu and colleagues [59] later reported that a ‘mixed liposome approach,’ in which gemcitabine and its co-drug were each encapsulated in separate liposomes, is advantageous in instances in which drug activity is dosage-sequence dependent. 

Liposomal gemcitabine has also been investigated in conjunction with gene therapy. Wang and colleagues [60] reported on the co-encapsulation of gemcitabine and anti-*KRAS* small interfering RNA (siRNA) in apolipoprotein E3-based liposomes. The combination of the siRNA, which downregulated the expression of the *KRAS* oncogene by the endogenous mechanism of RNA interference (RNAi), and gemcitabine improved pancreatic cancer cell apoptosis when compared with single-agent treatment. In a related study, it was reported that anti-*Mcl-1* siRNA co-delivery via cationic liposome could attenuate resistance to gemcitabine in pancreatic cancer [61]. A similar effect was observed when liposomal gemcitabine-treated lung cancer cells were pretreated with anti-*RRM1* siRNA [62], that targets the gene encoding a subunit of ribonucleotide reductase [63].

As an alternative to conventional drug loading, gemcitabine-conjugate was combined with cholesterol and phospholipids to form liposomes. The liposome inhibited tumor growth to a greater extent than free gemcitabine at less than 6 % of the normal dose, without systemic toxicity in a mouse model of pancreatic cancer [64].

## 3. Niosomes

Niosomes are formed by self-association of cholesterol and non-ionic surfactants in an aqueous phase. These nanostructures can be optimized for drug delivery by varying the composition, size, the number of lamellae, and surface charge. They are attractive for use in medicine, as they are biocompatible, non-immunogenic, highly stable, and have a long shelf-life [65]. Niosomes formulated from cholesterol, Span 60, and D-α-tocopheryl polyethylene glycol 1000 were loaded with gemcitabine and tocotrienols for improved efficacy in pancreatic cancer cells in vitro [66]. More recently, Saimi and co-workers [67] introduced aerosolized gemcitabine and cisplatin co-loaded niosome to treat lung cancer. The niosomes showed controlled release for both drugs for up to 24 h, and were found to be safe with growth inhibitory effects in non-small cell lung cancer.

## 4. Solid Lipid Nanoparticles

Solid lipid nanoparticles (SLNPs) are formulated from lipids that remain solid at physiological temperature and are stabilized by emulsifiers. SLNPs are biocompatible, biodegradable, can shield the encapsulated drug from harsh conditions [68] and have emerged as alternatives to liposomes as drug carriers. Nandini and co-workers [69] used a double emulsification technique to prepare gemcitabine loaded SLNPs from stearic acid, soy lecithin, and sodium taurocholate. The SLNPs showed controlled drug release and increased cellular uptake in several organs compared with the free drug. Affram and colleagues [70] studied the cytotoxic effect of gemcitabine-loaded SLNPs on pancreatic cells, in which the nanoparticle-associated drug demonstrated greater efficacy than the free drug. As with liposomes, SLNPs can also be ligand modified. Soni and co-workers [71] attached mannose to the surface of gemcitabine-loaded SLNPs to target the mannose-receptor on lung macrophages. 

Wang and co-workers [72] investigated the possibility of oral administration in mice with pre-established lung tumors. SLNPs loaded with a lipophilic amide prodrug of gemcitabine, 4-(*N*)-stearoyl gemcitabine, significantly inhibited tumor cell growth and angiogenesis, induced apoptosis and extended survival time. Studies have shown that the conjugation of fatty acids to the 4-*N* position of gemcitabine reduces sensitivity to deaminases [73]. The incorporation of the conjugate into nanoparticles provides further protection against deamination [74]. Lysosomes are reportedly beneficial for the attenuation of gemcitabine resistance by stearoyl gemcitabine SLNPs. It was put forward that the SLNP enters the cell via clathrin-mediated endocytosis and is fated for the lysosome where degradation of the SLNP allows for the release of the gemcitabine conjugate and its hydrolysis to free gemcitabine, and this is subsequently exported to the cytoplasm by nucleoside transporters [75].

## 5. Lipid/Calcium/Phosphate Nanoparticles

The lipid/calcium/phosphate (LCP) nanoparticle presents another avenue for drug delivery. Zhang and co-workers [76] precipitated phosphorylated gemcitabine within a calcium phosphate core, which was coated with a lipid bilayer to which PEG-chains were grafted at high density. Compared with free gemcitabine, in a mouse melanoma model, the LCP-loaded drug-induced apoptosis and reduced immunosuppression in the tumor microenvironment. As with other lipid-based carriers, co-drug delivery for an enhanced chemotherapeutic effect has also been explored with these nanoparticles. Gemcitabine and paclitaxel were co-loaded in a pegylated, cyclic RGD-modified LCP nanoparticle for targeted delivery to breast cancer cells. It was found that nanoparticles improved drug accumulation within tumors and nearly halted tumor growth with minimal general toxicity [77]. 

## 6. Nanostructured Lipid Carriers

Nanostructured lipid carriers (NLCs) are second-generation lipid nanoparticles that are prepared from solid and liquid lipids which give an amorphous solid matrix both at physiological and room temperature. They were developed to overcome the restrictions associated with solid-lipid nanoparticles, including low drug loading efficiencies and the risk of drug expulsion upon storage of the formulation [78]. Gemcitabine was conjugated to paclitaxel and formulated into NLCs modified with *N*-acetylglucosamine (NAG) to target glucose receptors on lung cancer cells [79]. The same group later introduced, also via NAG-modified NLC, a gemcitabine-paclitaxel drug-polymer conjugate with disulphide and ester linkages to exploit the tumor micro-environment conditions of high reducing potential and low pH for drug release [80]. In keeping with the idea of co-drug delivery, a hyaluronic acid-decorated NLC containing gemcitabine and baicalein gave encouraging results for the treatment of pancreatic cancer [81]. In a multi-drug delivery approach, a doxorubicin-gemcitabine prodrug co-loaded into NLCs with vincristine, showed excellent anti-tumor activity in lymphoma mouse xenografts in comparison with single drug-loaded NLCs and drug solutions [82]. 

## 7. Exosomes

Besides delivery via synthetic lipid-based nanostructures, gemcitabine can also be loaded into natural lipid vesicles such as exosomes. Exosomes are vesicles that are released from cells for the purpose of extracellular communication, function as natural carriers of a variety of biomolecules, and are favorable due to their high biocompatibility [83]. Gemcitabine was loaded into autologous exosomes for delivery to pancreatic tumors. Exosomes mediated a higher accumulation of the drug in tumor tissue and suppressed tumor growth without recurrence [84].

## 8. Lipid-Polymer Hybrid Nanoparticles 

Lipid-polymer hybrid nanoparticles (LPHNs), which aim to combine the advantages of lipid-based and polymeric nanostructures while overcoming their collective disadvantages [85], have been applied to the delivery of gemcitabine. A central composite design approach was used to fabricate an amalgamation of lipids and the co-polymer, poly(lactic-co-glycolic acid) (PLGA) for gemcitabine loading. The resulting LPHNs were 237 nm in size, had encapsulation efficiency of 45.2%, and a cumulative drug release of 62.3% at 24 h [86]. The same group applied LPHNs in vivo. The gemcitabine-loaded LPHNs exhibited longer circulation time and extended half-life when compared with the commercial drug [87]. As with liposomal gemcitabine delivery, LPHN-mediated gemcitabine delivery has also been combined with RNAi. A cationic ε-polylysine co-polymer was used to electrostatically associate with siRNA against the hypoxia-inducible factor 1α (HIF1α) gene that contributes to gemcitabine resistance when expressed at elevated levels in cancer cells. Gemcitabine was encapsulated within the hydrophilic core, and this was coated with a pegylated lipid bilayer to yield functional LPHNs [88].

## 9. Miscellaneous Lipid Nanoparticles

Dora and co-workers [89] prepared a novel micellar phospholipid complex of gemcitabine. In comparison with free gemcitabine, the complex displayed a sustained release pattern and high plasma stability. The complex performed favorably in toxicity studies with enhanced anticancer efficacy in a pancreatic cancer model. 

Bastiancich and colleagues [90] developed an injectable gel-like nanosystem made up of lipid nanocapsules loaded with a lauryl-gemcitabine conjugate for local treatment of glioblastoma. In vitro drug release was shown to be sustained and prolonged over a month. Furthermore, the system showed greater cytotoxic activity on U-87 MG glioma cells than the free drug and significantly reduced tumor size in vivo. Lipid nanocapsules containing gemcitabine were also shown to have monocyte-targeting properties, that can be useful for immunomodulation, in lymphoma and melanoma-bearing mice [91].

Gaudin and co-workers [92] nano precipitated a squalenoyl gemcitabine prodrug and a squalene-PEG conjugate to prepare nanoassemblies for convection-enhanced delivery to the brain. The nanoparticles improved treatment over free gemcitabine in an orthotopic model of glioblastoma multiform, both as a chemotherapeutic drug and a radiosensitizer. Similarly, squalenoyl-gemcitabine and edelfosine, an alkyl-lysophospholipid with proven anticancer activity, were associated with forming nanoparticles with high stability, high drug content, and anti-tumor activity in patient-derived osteosarcoma cells [93]. These nanoparticles were later suggested as a possible treatment for childhood osteosarcoma [94].

Recently, Comparetti and co-workers [95] introduced novel nanovesicles derived from the major components of the plasma membranes of neoplastic cells for the co-delivery of gemcitabine and paclitaxel. The nanoparticles exhibited high stability with enhanced cytotoxic effects in PANC-1 pancreatic cancer cells compared with conventional chemotherapy. Interestingly, the nanovesicles were capable of delivering antigenic material to antigen-presenting cells and could be useful for immunotherapy. 

All the above-mentioned lipid-based nanocarriers have shown the potential to be favorable nanocarriers of gemcitabine. Differences in drug loading capacity of the carriers may be influenced by physical characteristics such as size and charge [96]. Despite the advantages of such systems, there are still challenges that need to be addressed. Table 1 provides a summary of the advantages and disadvantages of the various delivery systems.

## 10. Conclusions

Within the past six years, significant developments have occurred in the field of lipid-mediated drug delivery, both with respect to the introduction of novel carriers and enhancement of existing ones. Recent developments, such as the advent of NLCs and niosomes, have provided more robust gemcitabine delivery systems and are responsive to modes of delivery other than systemic injection. The studies covered in this review highlight the merit of lipid-mediated gemcitabine delivery, especially with regards to overcoming the obstacles associated with conventional chemotherapy. Moreover, lipid-based nanostructures are amenable to its dosage with a co-drug and/or the delivery of its prodrug conjugates. Encouragingly, several studies have taken to in vivo models to provide proof of efficacy. Taken together, the publications under review suggest that lipid-based nano delivery platforms have the potential to revolutionize gemcitabine-mediated cancer treatment. The advent of a clinically viable gemcitabine nanoformulation is eagerly awaited.

## Figures and Tables

**Table 1:** Advantages and limitations of lipid-based nanoparticles as gemcitabine delivery agents.

Lipid-Based Nanoparticle	Advantages	Disadvantages
Liposomes	Biocompatible; biodegradable High loading capacity Flexibility of composition Targetable	Drug leakage High production cost Special storage conditions required
Niosomes	Biocompatible; non-immunogenic Osmotically active and stable Low production cost; long shelf-life	Drug leakage No human safety data available
Solid lipid nanoparticles	Biocompatible; biodegradable Low toxicity High bioavailability of drugs Targetable Amenable to large-scale production	Low drug loading efficiency Risk of drug expulsion upon storage No human safety data available
Lipid/calcium/phosphate nanoparticles	High encapsulation efficiency Efficient endosomal escape Sustained drug release Blood-brain barrier permeability Targetable	Complex structure and synthesis No human safety data available
Nanostructured lipid carriers	Biodegradable Increased drug loading Prevents drug expulsion Improved stability Targetable	No human safety data available
Exosomes	High biocompatibility High drug encapsulation efficiency Natural carriers Small size-tissue penetration Slightly negative zeta potential-extended circulation	Lack of standardized techniques for isolation and purification No human safety data available
Lipid-polymer hybrids	Robust delivery Well-defined release kinetics Good serum stability Targetable	Suboptimal drug loading and entrapment efficiency No human safety data available
Lipid nanocapsules	Biocompatible; small size Long-term stability Manufactured by low energy, organic, solvent-free process	No human safety data available
